# An Unusual Metastasis of a Transglottic Squamous Cell Carcinoma to the Forearm

**DOI:** 10.1155/2013/965329

**Published:** 2013-06-04

**Authors:** Abdullah Dafir Albeyatti, Richard Mark Kwasnicki, Derrick Siau, John de Carpentier

**Affiliations:** ^1^Department of ENT, Royal Preston Hospital, Sharoe Green, Lane, Preston PR2 9HT, UK; ^2^Department of Biosurgery and Surgical Technology, St Mary's Hospital, Praed Street, London W2 1NY, UK

## Abstract

*Introduction*. Each year around 2,200 people in the UK are diagnosed with laryngeal SCC (Office of National Statistics 2009). Compared to pharyngeal carcinoma, it is a highly curable disease with a survival rate of around 60% for all stages and all forms of treatment. *Case Presentation*. We present the case of a 60-year-old man with a previously treated T4 N2c transglottic squamous cell carcinoma (SCC), who developed an isolated swelling in the extensor compartment of his right forearm at 6 months after radical laryngectomy with bilateral neck dissection. Fine needle aspiration of the forearm lesion revealed SCC consistent with a metastasis from the laryngeal primary. MRI revealed that the lesion was confined to the muscle. Initial staging CT showed no distant metastases or signs of advanced disease, including no evidence of axillary nodal involvement. *Conclusion*. This case is therefore unusual, as one of only 2 cases reported in the scientific literature of isolated distant muscular metastasis from a laryngeal squamous cell carcinoma. We conclude that any muscular swelling, in the setting of previous head and neck malignancy, should be treated with a high degree of suspicion for metastasis and investigated promptly.

## 1. Introduction

Each year around 2,200 people in the UK are diagnosed with laryngeal SCC [1]. Compared to pharyngeal carcinoma, it is a highly curable disease with a survival rate of around 60% for all stages and all forms of treatment [2]. The site of the cancer within the larynx affects the prognosis. This is largely due to the stage at which the cancer causes symptoms. For example, a glottic tumour tends to affect the vocal cords early on and so presents with a persistent change in voice which is easily recognised by the patient. Conversely, a supraglottic tumour affects the vocal cords much later on, and the presenting symptom is often referred pain to the ear partly as a result of tumour extension to the hypopharynx. In addition, the lymphatics in the supraglottic larynx are more abundant to those in the true vocal cords, which makes early occult metastases to the cervical lymph nodes more common [3].

## 2. Case Presentation

A 60-year-old male presented to the ENT clinic in November 2008 with a two-month history of dysphonia. There were no other symptoms apart from lethargy. His past medical history included mild chronic obstructive airway disease which required no treatment. He was a cigarette smoker: 40 pack years and consumed approximately 20 units of alcohol per week mostly in the form of spirits.

On examination, he appeared systemically well, with no peripheral stigmata of chronic disease. Examination of the neck revealed bilateral cervical lymphadenopathy. There were multiple small (<2 cm), firm, well-circumscribed masses bilaterally which were mostly distributed within levels II-III of the neck. Flexible nasendoscopy performed in the clinic showed a fixed left hemilarynx. Examination under anaesthesia confirmed the findings of the flexible nasendoscopy, a large tumour of the left vocal cord crossing the anterior commissure to the right side along with slight subglottic extension.

**Figure 1:**
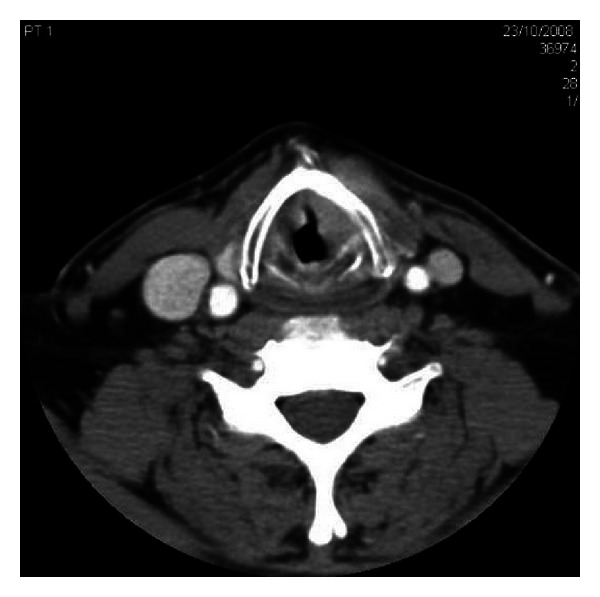
CT neck showing mass.

A staging CT scan of the head and neck was undertaken which showed a transglottic mass with significant local invasion and confirmed the bilateral enlarged cervical lymph nodes. No distant metastases were noted, including clear lung fields (Figure 1). 

Two weeks after presentation, total laryngectomy and bilateral modified radical neck dissections were performed. The histology revealed a 3.5 cm moderately differentiated (grade 2) SCC with microvascular, perineural, and thyroid cartilage invasion (T4). In addition, bilateral lymph nodes were positive for metastatic SCC with extracapsular spread evident on the left side (<6 cm greatest dimension). This cancer was therefore staged as T4N2c.

In January 2009 (2 months postoperatively) the patient attended a routine follow-up appointment. He was well and had no symptoms of local recurrence or distant metastatic spread; however, on examination a small parastomal lesion was seen. Prompt local excision of this was performed and histology showed a parastomal recurrence. As a consequence, chemoradiotherapy was started. 

**Figure 2:**
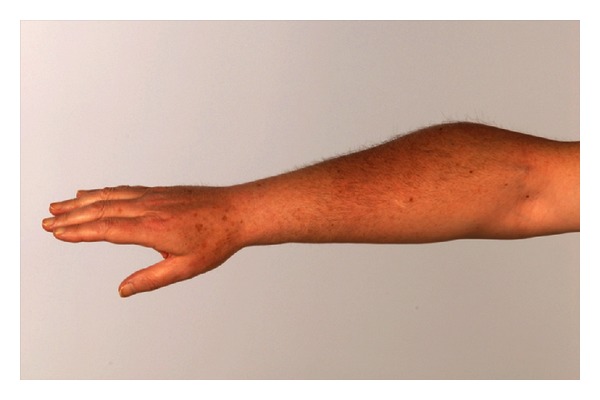
Right forearm—lesion on extensor compartment of right forearm measuring 10 × 7 cm.

Six months after the initial presentation, the patient returned with a 5-week history of a mild swelling in the extensor compartment of his right forearm (May 2009). The forearm was swollen and mildly tender, but there was no erythema, and nothing sinister was suspected (Figure 2). A short course of amoxicillin was started with no reduction in the size of the swelling. Orthopaedic review in July 2009 reported no loss of range of movement or injury to the elbow. During this time, the swelling had enlarged and was reported to be 10 × 7 cm. Subsequent fine needle aspiration of the lesion revealed metastatic SCC with similar morphology to the laryngeal primary cancer. An MRI of the forearm showed that the lesion was confined to the muscle. The patient was managed palliatively and unfortunately died 6 months later.

## 3. Discussion

Differential diagnoses regarding isolated limb swellings are plentiful and diverse and include cellulitis, trauma, postsurgical lymphoedema, DVT, and haematomas. These are all likely to be considered before malignancy. 72% of cases of laryngeal carcinoma occur over the age of 60 with the peak between 75 and 84 [1]. In addition to an elderly demographic, head and neck cancer patients often have other risk factors for vascular disease and coagulopathy, which may present as a limb swelling. These include smoking, alcohol abuse, malignancy, and self-neglect. Hence, swellings in the limbs of these patients are more likely to be attributed to clotting disorders and rarely thought to be directly related to their cancer. Only two cases have been reported of a laryngeal squamous cell carcinoma metastasising to a distant site, specifically the biceps femoris muscle [4] and rectus femoris muscle [5].

15–60% of our cardiac output supplies the skeletal muscles at rest and during exercise, respectively. If the body mass index is within the normal range, around 50% of the body is muscle. The musculature forms a significant proportion of our body and is well perfused; yet muscular metastases are uncommon.

Malignancies usually follow a step-wise pattern of progression, starting with local invasion, followed by lymphatic then haematogenous spread. With laryngeal cancer, local invasion of tissues often gives rise to the common presenting symptom of hoarseness (particularly with glottic tumours). Later, this may result in fixation of the vocal cords. Considering lymphatic drainage, the nodes of the internal jugular chain are most commonly involved in laryngeal cancer [6]. Lymphatic spread of a laryngeal cancer to a muscle in the forearm could only be a result of considerably aberrant flow. This is not impossible; surgical or other mechanical disruption of the lymphatic vessels can cause retrograde or collateral lymph drainage; however, this is most uncommon [7].

Successful distant metastasis relies on a series of steps: tumour transformation, dissociation and local invasion, intravasation, metastatic site stasis and extravasation, proliferation, and sustained growth. At each step there are various obstacles to overcome. Although the lymphatic and haematogenous systems are said to be in communication to an extent, survival of metastatic cancer cells in the blood stream appears far more difficult. In the 1970s, Fidler used radiolabelled cells to show that 99% of infused tumour cells are cleared from the blood within 24 hours [8]. This was consisted with a similar study by Weiss, who showed that less than 1% of disseminated tumour cells successfully metastasize [9]. As such, primary muscle malignancies are far more common than metastatic tumour deposits. Patients with muscular metastases in the absence of pleural or bony involvement may be candidates for local resection or radiotherapy in the interest of improving prognosis; however, most patients with a muscular metastasis die within a few months of diagnosis [10]. Local surgery or radiotherapy to relieve pain in the palliative setting may by indicated in suitable cases. 

## 4. Conclusion

This case illustrates why any distant swelling in a head and neck cancer patient should be treated with a high index of suspicion with regards to metastasis. Despite their initial innocuous appearance, imaging and histological investigations should be performed promptly to avoid any delay in diagnosis and treatment. Although muscular metastases are rare, they represent a very poor prognostic indicator judging by the life expectancy of these patients in the literature [10].
